# Long non-coding RNA LINC00426 contributes to doxorubicin resistance by sponging miR-4319 in osteosarcoma

**DOI:** 10.1186/s13062-020-00265-4

**Published:** 2020-07-03

**Authors:** Lulin Wang, Yi Luo, Yiquan Zheng, Lifeng Zheng, Wenxiang Lin, Zonglin Chen, Shichun Wu, Jinhong Chen, Yun Xie

**Affiliations:** 1 Department of Orthopaedics, Zhangzhou Affiliated Hospital of Fujian Medical University, No.59, Shengli Road West, Xiang Cheng District, Zhangzhou, 363000 Fujian China; 2 Department of Orthopaedic Trauma, Trauma Center of Fujian, The First Affiliated Hospital of Fujian Medical University, No.20, Chazhong Road, Taijiang District, Fuzhou, 350005 Fujian China

**Keywords:** LINC00426, miR-4319, Osteosarcoma, Doxorubicin resistance

## Abstract

**Background:**

LINC00426 is a newly identified long non-coding RNA (lncRNA) with unacknowledged biological roles. Here we set out to characterize the expression status of LINC00426 in osteosarcoma and understand its mechanistic involvement in incidence of doxorubicin (Dox) resistance.

**Methods:**

The relative expression of LINC00426 and miR-4319 was determined by real-time PCR. Cell viability and proliferation in response to LINC00426 silencing or miR-4319 over-expression was measured with CCK-8 kit and colony formation assay, respectively. The direct association between LINC00426 and miR-4319 was analyzed by pulldown assay with biotin-labelled probes.

**Results:**

LINC00426 was significantly up-regulated in Dox-resistant osteosarcoma (OS) both in vitro and in vivo, which intimately associated with unfavorable prognosis. SiRNA-mediated knockdown of LINC00426 remarkably compromised cell viability and proliferation in Dox-resistant OS cells, which accompanied with decrease of IC50 and activation of caspase-3. We further predicted and validated the regulatory effects of miR-4319 on LINC00426 expression. Simultaneously, we provided evidences in support of direct binding between LINC00426 and miR-4319 by pulldown assay. Reciprocally negative regulation was observed between LINC00426 and miR-4319 each other.

**Conclusion:**

Ectopic introduction of miR-4319 significantly surmounted the Dox resistance in OS cells, while miR-4319 inhibition in LINC00426-deficient cells greatly restore this phenotype. We uncovered the important contribution of LINC00426/miR-4319 to Dox resistance in osteosarcoma.

**Reviewers:**

This article was reviewed by Bo Liang and Sinan Zhu.

## Reviewers’ comments

Reviewers’ comments: (reviewer 1, Bo Liang)

This is a solid study with convincing results. My comments are amended below. 1. The writing of this paper should be improved. For example, the background of the abstract should include necessary information about LINC00426 and the aim of the current study. 2. More details are required to establish Dox resistance cell lines. This is essential information. 3. The figures are too crowded with many texts in the figure. Please refine. 4. The list of abbreviations is not complete. 5. The significance of this study should be expanded in the conclusion section.

Response:

1. The writing of this paper should be improved. For example, the background of the abstract should include necessary information about LINC00426 and the aim of the current study.

Reply:

We appreciate the reviewer’s kind suggestion and therefore we provided the background into LINC00426 and aim of our study in abstract section in page 3 line 2–3.

2. More details are required to establish Dox resistance cell lines. This is essential information.

Reply:

Thanks for your kind reminder, we have made an addition in the revised manuscript.

3. The figures are too crowded with many texts in the figure. Please refine.

Reply:

Thanks for your constructive suggestions, we have refined the figures in the revised version.

4. The list of abbreviations is not complete

Reply:

We apologize for this negligence and all abbreviations were listed in page 17 line 9–13 now.

5. The significance of this study should be expanded in the conclusion section

Reply:

We appreciated the reviewer’s invaluable suggestion and the significance of our study has been greatly highlighted in the conclusion section in page 17 line 3–6.

Reviewers’ comment: (reviewer 2, Sinan Zhu).

1. How was LINC00426 chosen in study from all other long non-coding RNAs? 2. What about the microRNAs could interact with this LINC00426? 3. Several statistical methods have been employed in the study. Method should be specified in the figure legends.

Response:

1. How was LINC00426 chosen in study from all other long non-coding RNAs?

Reply:

I really appreciate your concern about that. LINC00426 has been concerned in an occasional circumstance when we made the investigation of osteogenesis (a main research interests of our group), we ought to explore the functions and underlying mechanisms of LINC00426 in osteosarcoma. Previous research reported elevated LINC00426 was associated with osteosarcoma tumorigenesis and pulmonary metastasis [1], which indicated LINC00426 may play an oncogenic role in osteosarcoma, so we chose LINC00426 for further study.

[1] L. Xie, Z.H. Yao, Y. Zhang, D.Q. Li, F.D. Hu, Y.D. Liao, L. Zhou, Y.H. Zhou, Z.Y. Huang, Z.W. He, L. Han, Y.H. Yang, Z.Z. Yang, Deep RNA sequencing reveals the dynamic regulation of miRNA, lncRNAs, and mRNAs in osteosarcoma tumorigenesis and pulmonary metastasis, Cell Death Dis, 9 (2018).

2. What about the microRNAs could interact with this LINC00426?

Reply:

I really appreciate your concern about that. In Fig. 3, we first predicted the miRNA candidates by miRcode (http://www.mircode.org/mircode) and miR-4319 was chosen as the novel function-related miRNA for further study.

Luciferase reporter assay showed that miR-4319 could interact with LINC00426 (Fig. 3b, c); biotin-RNA pulldown assay indicated that miR-4319 could bind to LINC00426 directly (Fig. 3d, e, f); besides, LINC00426 acted as a “sponge” to negatively regulate the expression of miR-4319 in OS cells (Fig. 3g, h).

3. Several statistical methods have been employed in the study. Method should be specified in the figure legends.

Reply:

Thanks for your kind reminder, we have made an addition in the revised manuscript.

## Background

Osteosarcoma (OS) originates from the mesenchymal cells and is the most common primary bone malignancies affected children and adolescent, which is estimated to account for around 5% of all pediatric tumors [1]. The 5-year overall survival of OS based on the follow-up survey results is significantly improved in the last decade, which is greatly attributed to the advances in both of surgery and multiple combinational chemotherapy [2]. Acknowledged risk factors to this disease include bone dysplasia, TP53 mutant Li-Fraumeni syndrome and Rothmund-Thomson syndrome and frequent familiar genetic aberrance in chromosome 13q14 deletion, which consequently inactivates the retinoblastoma gene and links to high risk of osteosarcoma development [3]. Regular diagnosis of OS depends on X-ray and confirmative CT, PET, bone scan, MRI and surgical biopsy. The complete radical surgical resection is the treatment of choice in OS, which is applicable to as much as 90% of patients and clinical outcomes may be partially compromised by some complications including infection, prosthetic loosening and non-union, or local tumor recurrence. In this regard, current standard therapy of OS is combination of limb-salvage orthopedic surgery and high-dose of chemotherapeutic drugs such as methotrexate and leucovorin. Despite of the success of chemotherapy for osteosarcoma, the intrinsic and acquired drug resistances tremendously limited its clinical benefits [4]. The mechanistic elucidation of molecular events underlying the incidence and development of drug resistance will definitely guide our efforts toward more efficient and specific regimen for clinical management.

Long non-coding RNA (lncRNA) is defined as class of oligonucleotide longer than 200 nt without protein-coding potentials [5]. Accumulative evidences suggest the involvement of lncRNAs in multiple biological processing including cell differentiation, proliferation, apoptosis, metastasis, drug resistance and stemness. LINC00426 is a newly identified lncRNA with unacknowledged biological roles [6], which make it pretty novel to systematically address its function in the initiation and progression of osteosarcoma. The conventional view implicated that lncRNAs involve in gene transcription regulation via complexation with multiple factors including activator, suppressor, initiation protein, elongation protein and termination proteins in either gene-specific or -nonspecific ways [7]. In the recent decades, the reciprocal regulation between lncRNAs and microRNAs (miRs) is increasingly recognized, which antagonistically and consequently contribute to the fine-tune of target gene expression in distinct cell contexts. The sponge lncRNA concept is proposed to explain this phenomenon, and numerous sponging lncRNAs are identified so far participating in diverse physiological and pathological processing [8]. Here we focused on LINC00426 and its mechanistic involvements in osteosarcoma at both in vitro and in vivo levels. We further pursue the understanding into the molecular mechanism along the competing endogenous RNA direction. Our data will unmask the mysterious roles of LINC00426 in osteosarcoma, at least under the condition of Dox resistance.

miRs are increasingly documented to be critically involved in the tumor biology. Among which, the tumor suppressor functions of miR-4319 have been uncovered in array of human malignancies previously [9–11]. Here our data addressed an unrecognized role of miR-4319 in drug resistance in osteosarcoma, which might hold potential promises for therapeutic exploitation.

## Materials and methods

### Cell culture

Human OS cell lines (MG63, KHOS and U2OS) were obtained from the American Type Culture Collection (ATCC, NY, USA) and cultured in DMEM medium containing 15% fetal bovine serum (FBS, Gibco, MA, USA) and 1% penicillin/streptomycin (Invitrogen, MA, USA) at 37 °C in humidified CO_2_ (5%) incubator. The Dox resistance cell lines (MG63/DXR, KHOS/DXR) were established by serially increased concentration of Dox as described previously [12]. Briefly. the OS cells were continuously cultured in complete medium supplemented with 0.0035 μM Dox. The second (R2) generation was developed by continuous exposure of the corresponding R1 cells to 0.035 μM Dox. The third (R3) generation of Dox-resistant OS cells was generated by culturing the OS R2 cells in the continuous presence of 0.35 μM Dox. A cell line was considered as stable when the growth rate of the treated cells to a given Dox concentration was constant for at least 30 days.

### Cell transfection

Indicated cells were seeded into 6-well plate the day before transfection. Transfection was performed with Lipofectamine 3000 (Invitrogen, MA, USA) according to the manufacturer’s recommendation. The oligo sequences used in this study were provided as below:

si-LINC00426–1: 5′-GGCGCTATTTCGGCTCATTAT-3′;

si-LINC00426–2: 5′-GCGCTATTTCGGCTCATTATA-3′;

si-NC: 5′-TTCTCCGAACGTGTCACGT-3′;

miR-4319: 5′-UGCUCCCUGAGGACGUUAUAUGA-3′;

miR-NC: 5′-UGUGCAAAUCUAUGCAAAACUGA-3′.

### Real-time PCR

Total RNA was extracted from either cells or tissues with TriZol reagent (Invitrogen, MA, USA). The single-strand cDNA was prepared using the PrimeScript RT Reagent Kit (Takara, Dalian, China). The relative expression of LINC00426 and miR-4319 was determined with SYBR Green MasterMix Kit (Roche, Basel, Switzerland) on ABI PRISM 7900 HT System (Applied Biosystems, CA, USA). GAPDH and U6 were employed as reference control. All primer sequences were listed as follows:

miR-4319 RT prime: 5′-GTCGTATCCAGTGCAGGGTCCGAGGTATTCGCACTGGATACGACGTGGCT-3′;

U6 RT prime: 5′-GTCGTATCCAGTGCAGGGTCCGAGGTATTCGCACTGGATACGACAAAATATGGAA-3′;

LINC00426 qRT-PCR Forward: 5′-CAAGAAGACAGGGACAAGC-3′;

LINC00426 qRT-PCR Reverse: 5′-ACTGAGTACCCAGCCAAAG-3′;

GAPDH qRT-PCR Forward: 5′-CATGAGAAGTATGACAACAGCCT-3′;

GAPDH qRT-PCR Reverse: 5′-AGTCCTTCCACGATACCAAAGT-3′;

miR-4319 qRT-PCR Forward: 5′-CACCCAGAGCAAAGCCAC-3′;

miR-4319 qRT-PCR Reverse: 5′-GTGCAGGGTCCGAGGT-3′;

U6 qRT-PCR Forward: 5′-TGCGGGTGCTCGCTTCGGCAGC-3′;

U6 qRT-PCR Reverse: 5′-GTGCAGGGTCCGAGGT-3′.

### Cell counting kit (CCK)-8 assay

CCK-8 kit (Dojindo, Kumamoto, Japan) was used to measure cell viability. Briefly, 4000 indicated cells/well were seeded into 96-well plates in triplicated and subjected to specified transfection for 48 h. CCK-8 working solution was added and allowed for reaction for 2 h at 37 °C. Absorption at 450 nm was determined with microplate reader (Biotek, VT, USA) and relative cell viability was calculated accordingly.

### Colony formation assay

Cell proliferation was evaluated by colony formation assay. The transfected cells (1000 cells) were prepared into 6-well plate in triplicate and continuously cultured for 2 weeks. The former colonies were fixed with PFA (4% in PBS) first and stained with crystal violet (0.05% in PBS) for 10 min. Colony number was counted under light microscope and representative images were captured and presented.

### Caspase-3 activity assay

The indicated cells were collected by trypsin digestion and caspase-3 activity was determined with Cells were treated with different concentrations of MTX, ADM or DDP for 72 h and then collected with trypsin. The caspase-3 activity was measured with Caspase-3 Activity Assay Kit (Merck, MO, USA) according to the manufacturer’s manual. Briefly, cell lysate was prepared in the supplied lysis buffer and substrate was added for 2 h-incubation at 37 °C in the dark. The absorption at 405 nm was recorded with microplate reader and relative caspase-3 activity was calculated accordingly.

### Luciferase reporter assay

LINC00426-driven luciferase reporter plasmids were constructed by PCR-Restriction Digestion-Ligation method. The mutant was generated by mutagenesis PCR method. The primer sequences used for PCR were provided as below. Both luciferase plasmids and miR-4319 were co-transfected into MG63/DXR and KHOS/DXR cells in triplicate in 6-well plate (1◊10^6^ cells/well). The cell lysates were collected and dual luciferase activities were determined with Dual-Luciferase Reporter Assay System (Promega, WI, USA).

LINC00426 PCR Forward: 5′-AATTCTCGAG ACTCGGCCATGAAAGTC-3′;

LINC00426 PCR Reverse: 5′-AATTGCGGCCGC TGTCTTCCAGTAAGACTTTA-3′;

LINC00426 mutagenesis PCR Forward: 5′-CACCACTTGTCTCAGAGGAA-3′;

LINC00426 mutagenesis PCR Reverse: 5′-TTCCTCTGAGACAAGTGGTG-3′.

### Pull-down assay

RNA was completely removed by RNase A incubation. Biotin-labeled probes were incubated with cell lysates from indicated cells (2.5 × 10^7^) for one hour at room temperature. Pulldown assay was performed with streptavidin-coated agarose beads (Invitrogen, MA, USA) and enriched RNA specimen was reversely transcribed into cDNA as described previously. The relative abundance of target transcript was determined by real-time PCR. The probe sequences used in this study were provided as below:

Sense DNA probe: 5′-Biotin-GTCAGGACACAGCAAATGGGGGATCT-3′;

Antisense DNA probe: 5′- Biotin-AGATCCCCCATTTGCTGTGTCCTGAC-3′;

Bio-miR-NC: 5′-Biotin-UGUGCAAAUCUAUGCAAAACUGA-3′;

Bio-miR-4319: 5′-Biotin-UGCUCCCUGAGGACGUUAUAUGA-3′.

### Statistical analysis

Results were presented as the mean values ± SD unless specified and analyzed with two-tailed *t*-test, one- or two-way ANOVA with a Bonferroni post hoc test. A value of *p* < 0.05 was considered significantly different.

## Results

### Expression level of LINC00426 in OS closely correlated with doxorubicin resistance and clinical outcomes

**Fig. 1:**
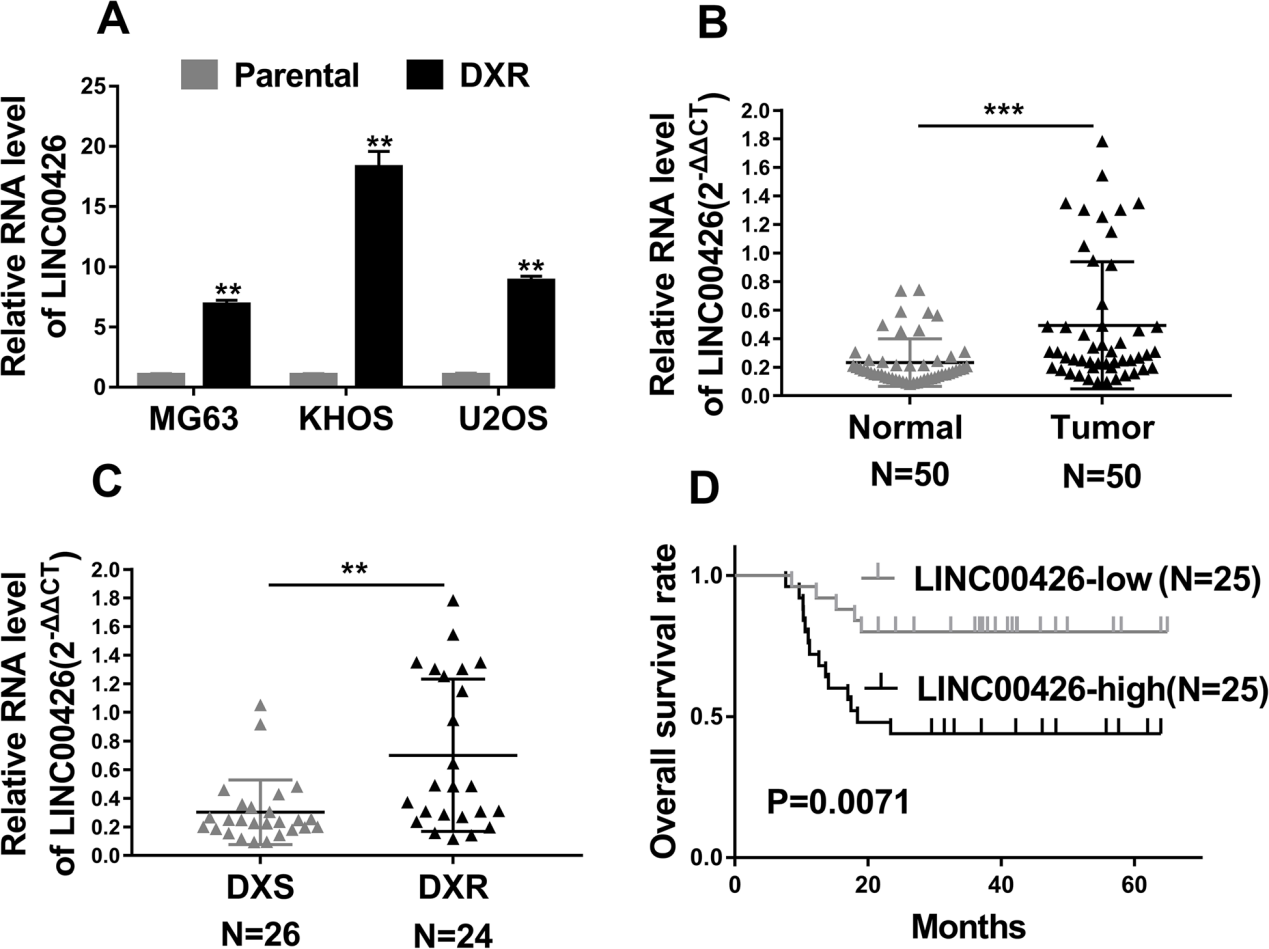
Expression level of LINC00426 in OS closely correlated with doxorubicin resistance and clinical outcomes. **a** The RNA level of LINC00426 in the three pairs of doxorubicin-resistant (DXR) and doxorubicin-sensitive (Parental) OS cell lines was detected by qRT-PCR. **b** qRT-PCR analysis of LINC00426 RNA level in 50 normal adjacent tissues and osteosarcoma tumors (shown as 2^-ΔΔCT^). **c** LINC00426 expression significantly increased in specimens of OS patients who were doxorubicin-resistant (DXR, *n* = 24) compared to those who were doxorubicin-sensitive (DXS, *n* = 26). **d** Kaplan-Meier survival rates for OS patients with low (*n* = 25) and high (n = 25) LINC00426 expression. Log-rank test for D, Student’s t-tests for others. *P < 0.05; **P < 0.01; ***P < 0.001

We first determined the relative expression of LINC00426 in three established doxorubicin-resistant OS cell lines in comparison with the sensitive parental cells. As shown in Fig. 1a, dramatic increases of LINC00426 transcript were observed in all of three cell lines, which indicated the intimate linkage between high abundance of LINC00426 with doxorubicin resistance at least in vitro. The up-regulation of LINC00426 in OS patients was further confirmed in 50 paired OS clinical samples (Fig. 1b). Notably, the significant higher level of LINC00426 was noticed in doxorubicin-sensitive patients compared with the resistant counterparts (Fig. 1c). Consistently, the Kaplan-Meier analysis demonstrated that low expression of LINC00426 significantly associated with better clinical outcomes (Fig. 1d). Therefore, both in vitro and in vivo data suggested that LINC00426 closely correlated with doxorubicin resistance and clinical survival.

### LINC00426 inhibition re-sensitized OS cells to doxorubicin

**Fig. 2:**
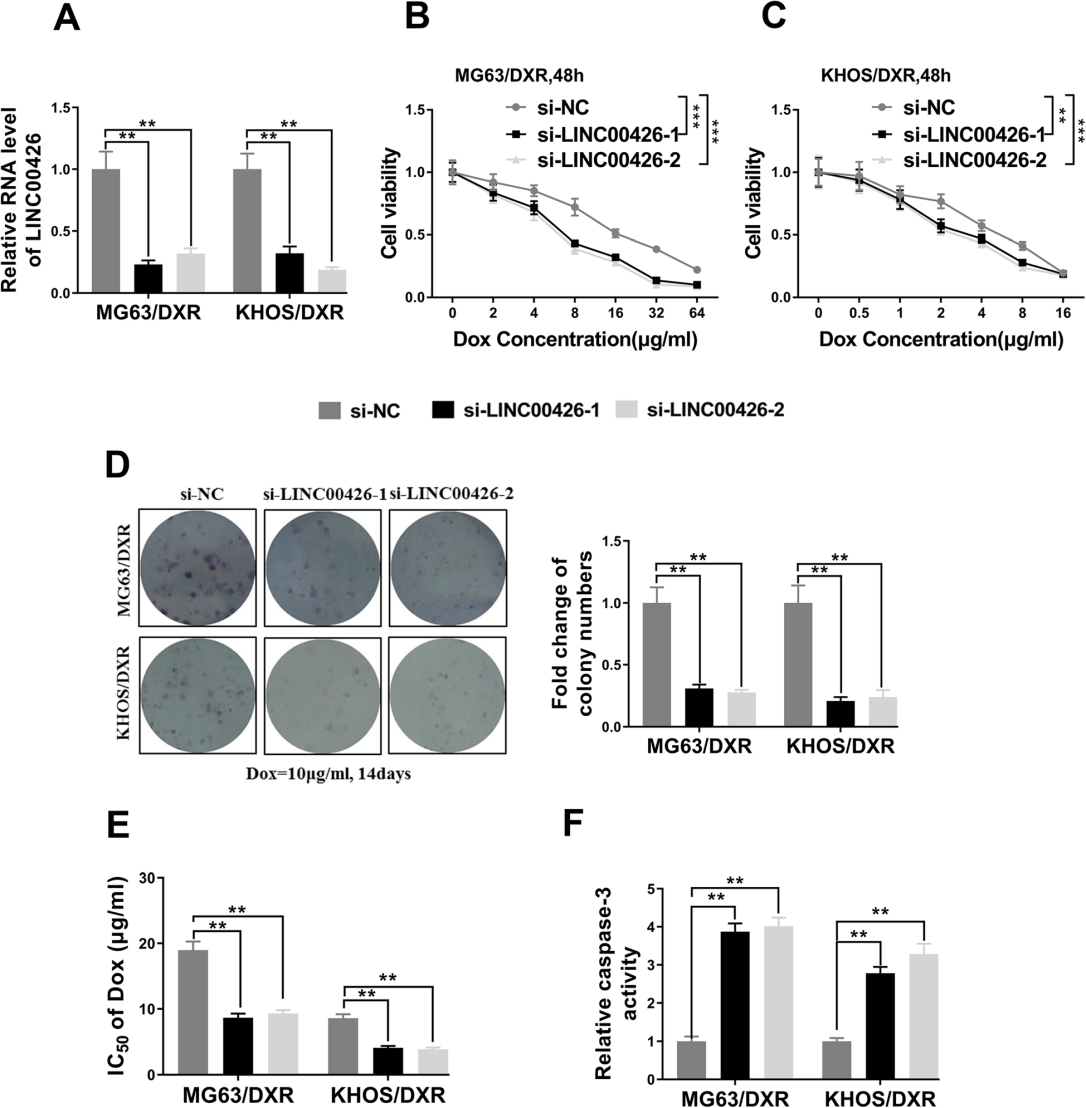
LINC00426 inhibition re-sensitized OS cells to doxorubicin. **a** The relative expression level of LINC00426 in doxorubicin-resistant MG63 (MG63/DXR) and KHOS (KHOS/DXR) cells transfected with LINC00426 siRNAs (si-LINC00426–1, si-LINC00426–2) or negative control siRNA (si-NC) was detected by qRT-PCR. **b** and **c** Cell counting kit-8 assays were performed to examine the cell proliferation rate of MG63/DXR and KHOS/DXR cells in response to doxorubicin after LINC00426 knockdown. **d** Clone formation assays were performed to examine cell vitality of MG63/DXR and KHOS/DXR cells to doxorubicin exposure (10 μg/ml, 14 days) after LINC00426 knockdown. **e** IC_50_ values of MG63/DXR and KHOS/DXR cells transfected with LINC00426 siRNAs (si-LINC00426–1, si-LINC00426–2) greatly decreased compared with those of the negative control siRNA (si-NC) group. **f** Caspase-3 activity assays were performed to examine cell apoptosis of MG63/DXR and KHOS/DXR cells to doxorubicin exposure (0.1 μg/ml, 48 h) after LINC00426 knockdown. The data represent the mean ± SD. Two-way ANOVA for B and C, Student’s t-tests for others. *P < 0.05; **P < 0.01; ***P < 0.001

Our previous results indicated the causal relation between high abundance of LINC00426 with doxorubicin resistance and clinical outcomes, which prompted us to investigate the potential benefits of LINC00426 inhibition in this scenario. Two independent siRNAs were employed to specifically silenced LINC00426 in both MG63/DXR and KHOS/DXR cells, and the knockdown efficiency was experimentally confirmed by real-time PCR (Fig. 2a). Cell viability measured with CCK-8 kit unambiguously was greatly compromised by LINC00426 depletion in both cells (Fig. 2b, c). Furthermore, the cell proliferation was significantly suppressed by LINC00426-specific siRNAs in both cell lines as well in comparison with scrambled negative control (Fig. 2d). Most importantly, the relative sensitivity to doxorubicin was markedly restored upon LINC00426 knockdown as indicated by the decrease of IC50 value (Fig. 2e), which was accompanied by the remarkable increase of caspase-3 activity in LINC00426-deficient cells (Fig. 2f). Therefore, our data clearly demonstrated that LINC00426-silencing fundamentally contributed to the re-sensitization of resistant cells to doxorubicin treatments.

### LINC00426 acted as a “sponge” of miR-4319 in OS cells

**Fig. 3:**
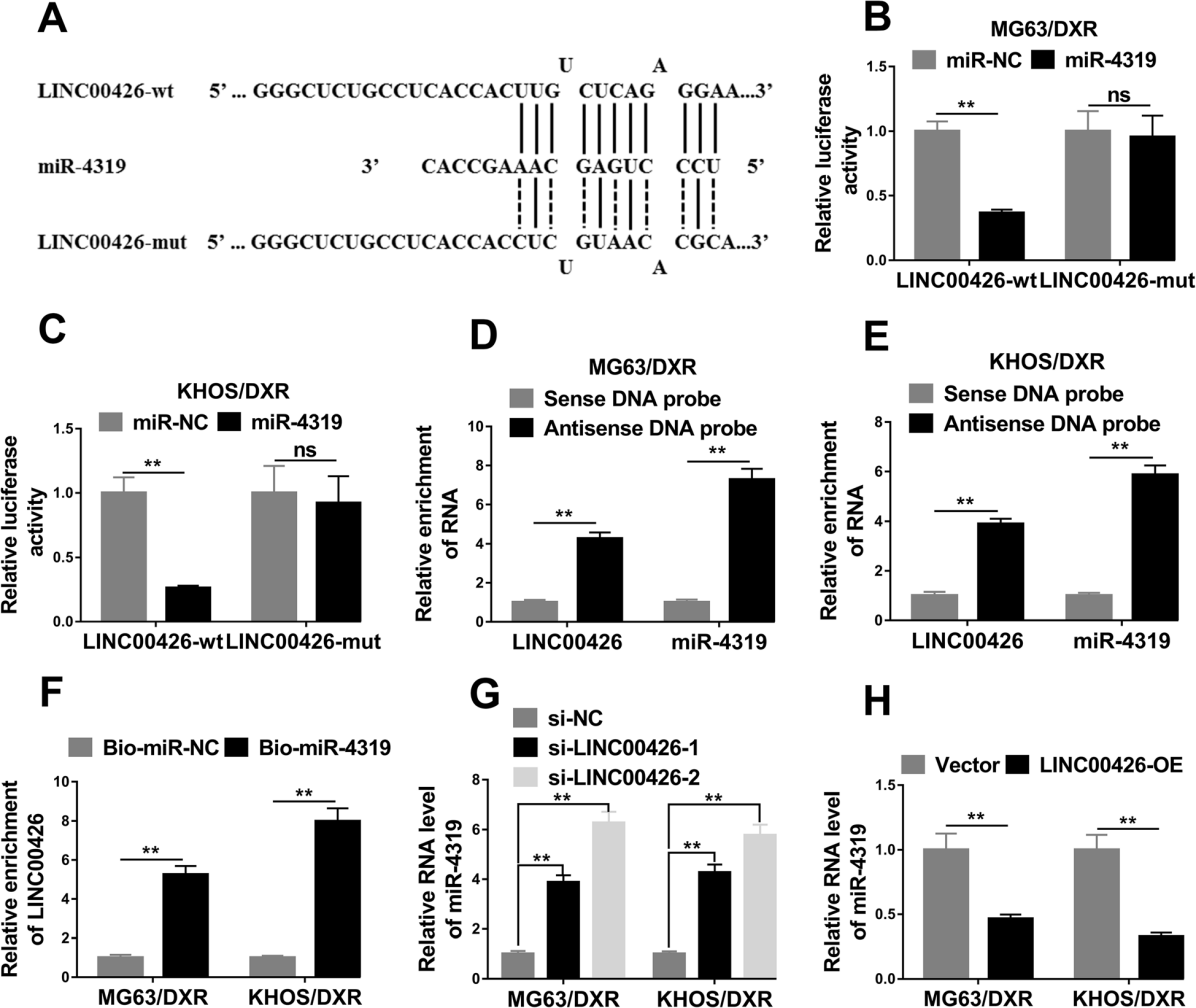
LINC00426 acted as a “sponge” of miR-4319 in OS cells. **a** The prediction for miR-4319 binding sites on LINC00426 transcript and schematic of luciferase reporter vector constructs LINC00426 wild-type (LINC00426-wt) and the miR-4319-binding-site mutated (LINC00426-mut) one. **b** and **c** The luciferase activities in MG63/DXR and KHOS/DXR cells co-transfected with miR-4319 or miR-NC mimics and luciferase reporters containing LINC00426-wt or LINC00426-mut. Data are presented as the relative ratio of hRluc luciferase activity to hluc+ luciferase activity. **d** and **e** Lysates from MG63/DXR and KHOS/DXR cells were incubated with in vitro-synthesized biotin-labeled sense or antisense DNA probes against LINC00426 for biotin pull-down assay, followed by qRT-PCR analysis to examine LINC00426 and miR-4319 levels. **f** Detection of LINC00426 in biotinylated miRNA/target complex by real-time RT-PCR. The relative level of LINC00426 in the complex pulled down by using biotinylated miR-4319 (Bio-miR-4319) was compared to that of the complex pulled down by using the biotinylated control random RNA (Bio-miR-NC) in MG63/DXR and KHOS/DXR cells. **g** The relative expression levels of miR-4319 in MG63/DXR and KHOS/DXR cells transfected with LINC00426 siRNAs (si-LINC00426–1, si-LINC00426–2) or negative control siRNA (si-NC) were detected by qRT-PCR. **h** The relative expression levels of miR-4319 in MG63/DXR and KHOS/DXR cells transfected with LINC00426 overexpression plasmid (LINC00426-OE) or empty vector (Vector) were detected by qRT-PCR. The data represent the mean ± SD. Student’s t-tests. *P < 0.05; **P < 0.01. ns = not significant

To better understand the mechanistic involvements of LINC00426 in osteosarcoma, we next employed the bioinformatic tool miRcode (http://www.mircode.org/mircode) to predict and discover the possible target microRNA of LINC00426. Number of microRNAs were predicted with affinity to LINC00426 and miR-4319 was the top one in the list. The alignment between miR-4319 and either wild-type or mutant LINC00426 sequences were illustrated in Fig. 3a. To interrogate the potentially regulatory effects of miR-4319 on LINC00426, we constructed LINC00426-fused luciferase reporter plasmids. Co-transfection with miR-4319 greatly suppressed the LINC00426-driven luciferase activity in MG63/DXR cells in comparison with negative control, which was readily abolished by the mutation introduced into the putative recognizing site in LINC00426 transcript (Fig. 3b). Similar observation was confirmed in KHOS/DXR cells as shown in Fig. 3c. The binding between LINC00426 and miR-4319 was directly analyzed by pulldown assay with biotin-labelled probes. As shown in Fig. 3d and e, remarkable enrichments of both LINC00426 and miR-4319 were observed with antisense DNA probe other than sense DNA probe in both cell lines. Reciprocally, LINC00426 transcripts were significantly enriched in the miR-4319 pulldown complex in comparison with biotin-labelled negative control (Fig. 3f). We further noticed that endogenous miR-4319 was greatly up-regulated in LINC00426-deficient cells and inhibited by exogenously introduced LINC00426 expression (Fig. 3g, h). Taken together, we provided evidences in support of the sponging function of LINC00426 against miR-4319 in osteosarcoma.

### MiR-4319 acted as an inhibitor in the OS doxorubicin resistance

**Fig. 4:**
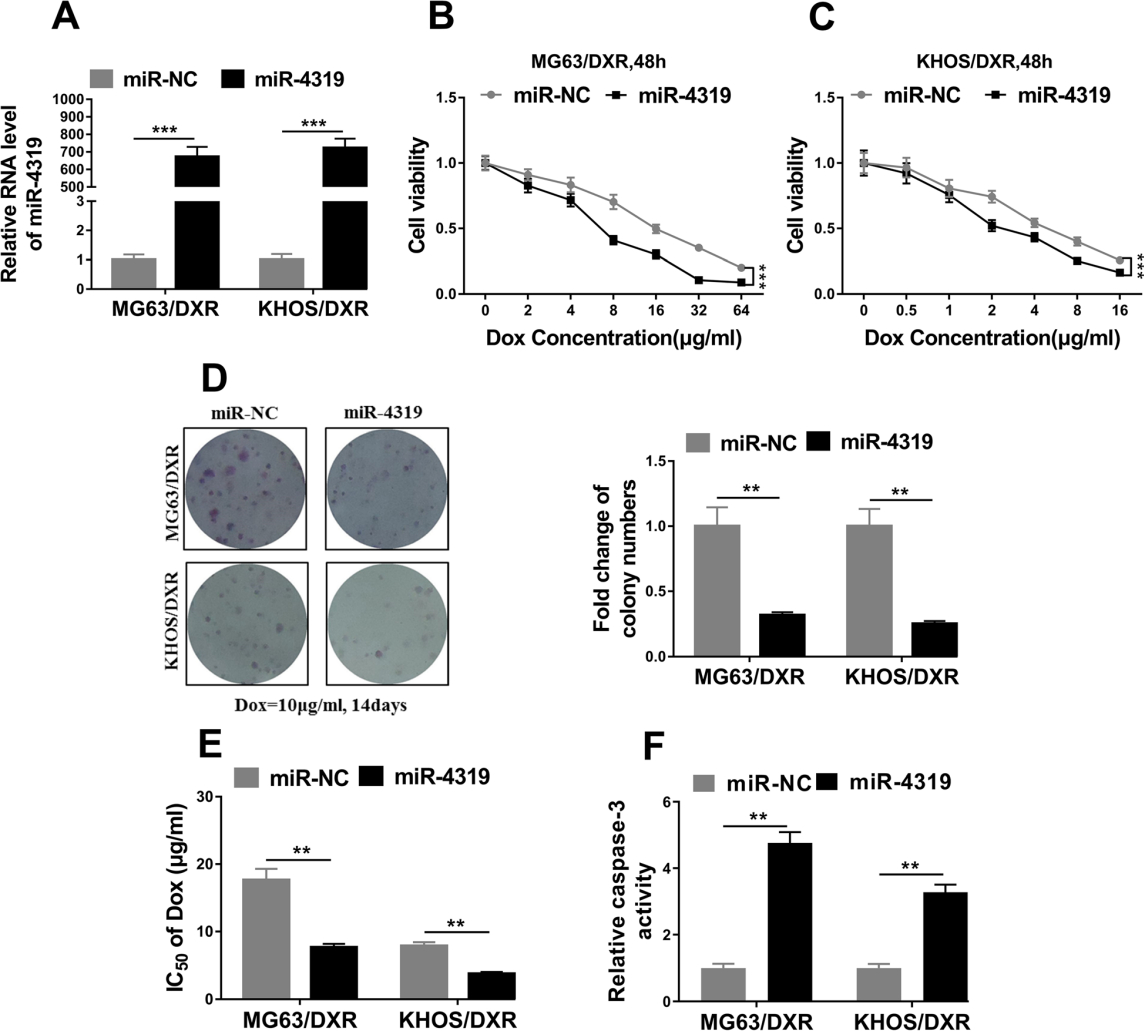
MiR-4319 acted as an inhibitor in the OS doxorubicin resistance. **a** The relative expression level of miR-4319 in doxorubicin-resistant MG63 (MG63/DXR) and KHOS (KHOS/DXR) cells transfected with miR-4319 mimics (miR-4319) or negative control miRNA (miR-NC) was detected by qRT-PCR. **b** and **c** Cell counting kit-8 assays were performed to examine the cell proliferation rate of MG63/DXR and KHOS/DXR cells in response to doxorubicin after miR-4319 overexpression. **d** Clone formation assays were performed to examine cell vitality of MG63/DXR and KHOS/DXR cells to doxorubicin exposure (10 μg/ml, 14 days) after miR-4319 overexpression. **e** IC_50_ values of MG63/DXR and KHOS/DXR cells transfected with miR-4319 mimics greatly decreased compared with those of the negative control miRNA (miR-NC) group. **f** Caspase-3 activity assays were performed to examine cell apoptosis of MG63/DXR and KHOS/DXR cells to doxorubicin exposure (0.1 μg/ml, 48 h) after miR-4319 overexpression. The data represent the mean ± SD. Two-way ANOVA for B and C, Student’s t-tests for others. *P < 0.05; **P < 0.01; ***P < 0.001

Our previous data suggested the negative regulation of LINC00426 on miR4319 expression in OS, which might functionally mediate the pathological roles of LINC00426 in this disease. To clarify this possibility, we forced over-expression of miR-4319 in MG63/DXR and KHOS/DXR cells (Fig. 4a). The ectopic expression of miR-4319 significantly inhibited cell viability as indicated by the CCK-8 results obtained from both cell lines (Fig. 4b, c). Consistently, cell proliferation was compromised as well by miR-4319 overexpression (Fig. 4d). The sensitivity to doxorubicin was also greatly improved by miR-4319 over-expression as implicated by the decrease in IC50 value of Dox (Fig. 4e). Again, the relative caspase-3 activity was remarkably restored by ectopic introduction of miR-4319 (Fig. 4f). In summary, our data suggested that miR-4319 acted as an inhibitor against doxorubicin resistance occurrence in OS.

### LINC00426 promoted OS doxorubicin resistance by sponging miR-4319

**Fig. 5:**
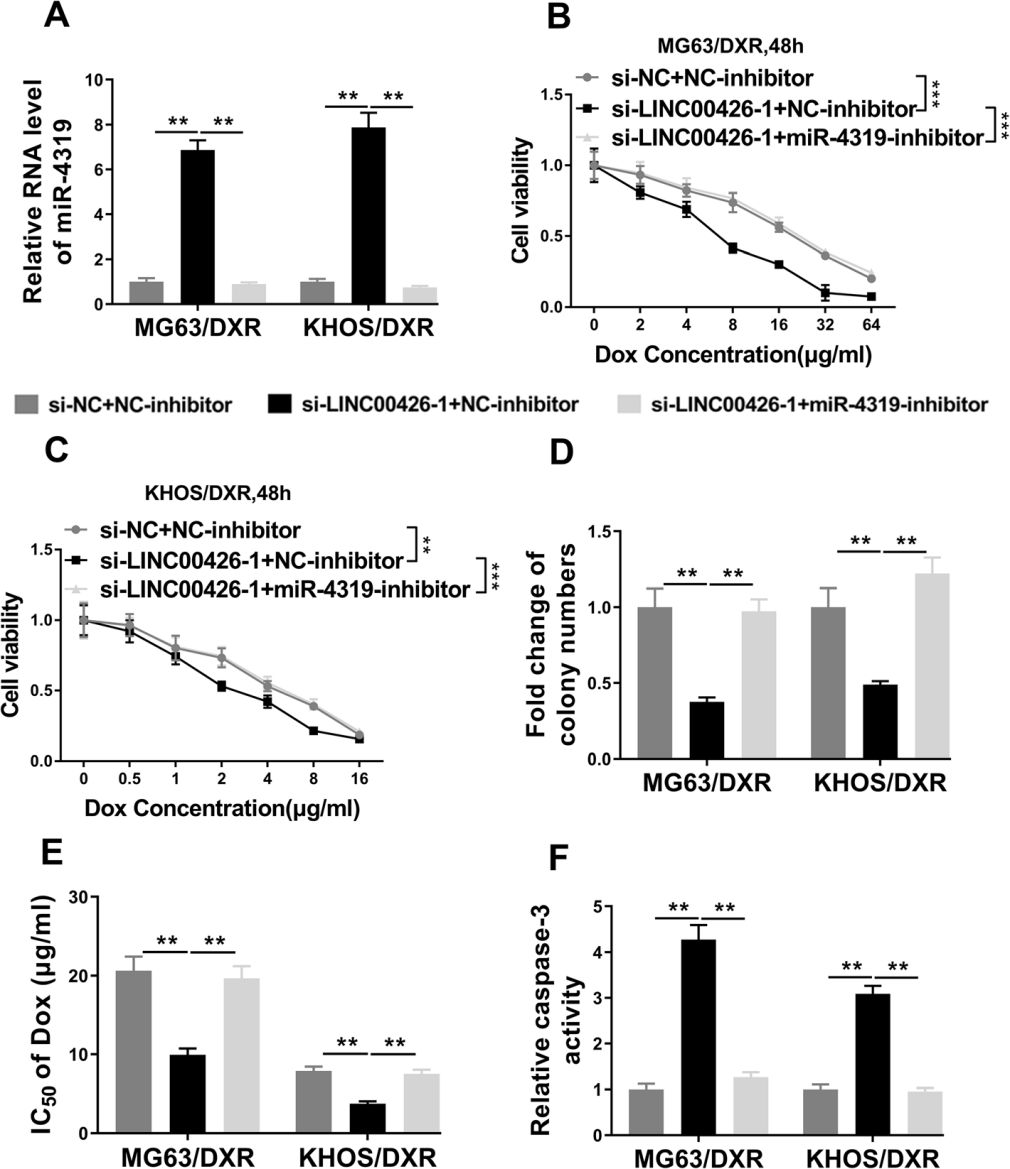
LINC00426 promoted OS doxorubicin resistance by sponging miR-4319. **a-f** MG63/DXR and KHOS/DXR cells were co-transfected with si-NC + NC-inhibitor, si-LINC00426–1 + NC-inhibitor or si-LINC00426–1 + miR-4319-inhibitor. **a** The RNA levels of miR-4319 in these co-transfected MG63/DXR and KHOS/DXR cells were determined by qRT-PCR. **b** and **c** Cell counting kit-8 assays were performed to examine the cell proliferation rate of these co-transfected MG63/DXR and KHOS/DXR cells in response to doxorubicin. **d** Clone formation assays were performed to examine cell vitality of these co-transfected MG63/DXR and KHOS/DXR cells to doxorubicin exposure (10 μg/ml, 14 days). **e** IC_50_ values of these co-transfected MG63/DXR and KHOS/DXR cells. **f** Caspase-3 activity assays were performed to examine cell apoptosis of these co-transfected MG63/DXR and KHOS/DXR cells to doxorubicin exposure (0.1 μg/ml, 48 h). The data represent the mean ± SD. Two-way ANOVA for B and C, Student’s t-tests for others. *P < 0.05; **P < 0.01; ***P < 0.001

Although we demonstrated the sponging function of LINC00426 against miR-4319 and improving effects of miR-4319 on Dox resistance in OS cells, whether miR-4319 predominantly contributed to doxorubicin resistance downstream LINC00426 was still to be addressed. To this end, we specifically inhibited miR-4319 in the LINC00426-deficient cells, the relative expression of miR-4319 in this scenario was validated by real-time PCR (Fig. 5a). The compromised cell viability elicited by LINC00426 deficiency was almost completely restored by miR-4319-inhibitor in both MG63/DXR and KHOS/DXR cells (Fig. 5b, c). Similarly, the cell proliferation inhibited by si-LINC00426 was stimulated by co-transfection with miR-4319-inhibitor (Fig. 5d). Further analysis demonstrated that miR-4319 inhibition in the LINC00426-silenced cells remarkably increased IC50 value of Dox, which suggested the re-occurrence of drug resistance under this condition (Fig. 5e). Consistently, the activation of caspase-3 was dramatically suppressed by miR-4319-inhibitor in both cell lines (Fig. 5f). Taken together, our data indicated that LINC00426 promoted Dox resistance in OS via sponging miR-4319.

## Discussion

The critical contributions of lncRNAs in human malignancies were increasingly acknowledged [13]. In this study, we concentrated on the mechanistic involvements of LINC00426 in osteosarcoma. Our results first uncovered the aberrant over-expression of LINC00426 in doxorubicin-resistant OS cell lines in comparison with parental counterparts. In vivo analysis manifested the general up-regulation of LINC00426 in OS tumor and specifically elevated in the Dox un-responding patients. Higher LINC00426 abundance intimately associated with unfavorable prognosis as well, which might be due to the poorer response to Dox treatment. Employment of LINC00426-specific siRNAs in Dox-resistant cells significantly compromised both cell viability and proliferation, which was accompanied with improvement in response to Dox treatment and restoration of caspase-3 activation. With aid of bioinformatics tool, we predicted and experimentally validated that miR-4319 as direct target of LINC00426. Exogenous miR-4319 greatly suppressed the LINC00426-driven luciferase activities, and this inhibitory effect was readily abolished by the mutation introduced into LINC00426 transcript. The direct binding between LINC00426 and miR-4319 was experimentally validated with pulldown assay, wherein significant enrichment of both LINC00426 and miR-4319 was observed in biotin-labelled anti-sense DNA probe and vice versa. The reciprocal regulation between LINC00426 and miR-4319 was also confirmed in the Dox resistant cell lines. More importantly, ectopic supplementation with miR-4319 greatly inhibited cell viability and proliferation, which was accompanied with decrease of IC50 value of Dox and activation of caspase-3. Oppositely, inhibition of miR-4319 remarkably restored both cell viability and proliferation in LINC00426-deficient cells. Simultaneously, co-transfection with miR-4319-inhibitor led to re-occurrence of Dox resistance as indicated by the increase of IC50 value and decrease of caspase-3 activation. In summary, our data elucidated that high abundance of LINC00426, via negative regulation of miR-4319 expression, consequently contributed to the Dox resistance incidence in OS.

Drug resistance was inevitable for cancer chemotherapy and number mechanisms underlying this phenomenon were uncovered so far [14]. The fundamental contribution of lncRNAs in this scenario has increasingly received attentions from research community. For instance, Bai et al. showed that lncRNA LOXL1-AS1/miR-let-7a-5p/EGFR-related signaling modulated the Dox resistance in prostate cancer cell DU-145 [15]. Cai et al. reported that lncRNA GBCDRlnc1 provoked chemoresistance through activation of autophagy in gallbladder cancer [16]. In myeloid leukemia cells, Yang et al. proposed that lncRNA linc00239 underlay the malignancies and chemoresistance against Dox by PI3K/Akt/mTOR signaling over-activation [17]. Knockdown assay performed by Dong et al. demonstrated that lncRNA HOXA-AS2 deficiency suppressed the chemoresistance through miR-520c-3p/S100A4 pathway [18]. Similar conclusion was drawn in colorectal cancer by Zhu et al. manifested that lncRNA XIST silencing greatly compromised the Dox resistance via increase of miR-124 and decrease of SGK1 [19]. Zhu et al. further disclosed that Fibronectin-1 regulated by lncRNA OIP5-AS1/miR-200b-3p underlined the Dox resistance in osteosarcoma cells [20]. Zhou et al. suggested that Dox resistance in osteosarcoma provoked by lncRNA SNHG12 might be attributed to miR-320a/MCL1 signaling axis [21]. Here we for the first time provided evidence in support of the novel involvements of LINC00426 in the drug resistance in osteosarcoma, which predominantly mediated by negative regulation of miR-4319.

During the incidence of Dox resistance, number of microRNAs were identified to play critical roles in variety of human cancers. Liu et al. recently demonstrated the imposed Dox resistance in gastric cancer by exosomal transfer of miR-501 to specifically target BLID. MiR-134 was characterized to regulate resistance to Dox in human breast cancer cells via down-regulation of ABCC1 [22], while miR-132 and miR-212 were identified by Xie et al. to mediated Dox resistance through suppression of PTEN-AKT/NF-κB pathway [23]. In hepatocellular carcinoma, Tian et al. suggested that miR-760 inhibited Dox resistance via targeting Notch1/Hes1-PTEN/Akt signaling [24]. In osteosarcoma, several miRs were proposed to link with the occurrence of Dox resistance. Lin et al. firstly demonstrated that miR-184/BCL2L1 signaling axis contributed to Dox resistance in osteosarcoma [25]. Zhou et al. subsequently suggested that lncRNA SNHG12 modulated Dox resistance through miR-320a/MCL1 signaling [21]. The study performed by Zhang et al. indicated that miR-301a regulated Dox resistance via modulating AMP-activated protein kinase alpha 1 [26]. More recently, an intriguing investigation conducted by Wang et al. showed that lncRNA CTA significantly sensitized osteosarcoma cells to Dox treatment via inhibition of autophagy [27]. Noting worthily, our data highlighted the critical role of miR-4319 in the Dox resistance in osteosarcoma, which might hold great therapeutic promises for the future exploitations.

## Conclusion

In summary, we uncovered the mechanistic contribution of LINC00426/miR-4319 to Dox resistance in osteosarcoma, which warranted further investigation and translational developments. Our study has, for the first time, unraveled the important clinical roles of LINC00426 in osteosarcoma response to Dox and identified its competing miR, which has shed new light on the incidence of Dox resistance in this disease and offered new opportunity for therapeutic intervention.

## Data Availability

All data generated or analyzed during this study are included in this published article.

## Abbreviations

Dox: Doxorubicin
OS: Osteosarcoma
PCR: Polymerase chain reaction
CCK-8: Cell counting kit-8
CT: Computed tomography
PET: Positron emission computed tomography
MRI: Magnetic resonance imaging
lncRNA: Long non-coding RNA
miRs: MicroRNAs
ATCC: American Type Culture Collection
FBS: Fetal bovine serum
PFA: Paraformaldehyde
PBS: Phosphate buffer saline
